# Using web search queries to monitor influenza-like illness: an exploratory retrospective analysis, Netherlands, 2017/18 influenza season

**DOI:** 10.2807/1560-7917.ES.2020.25.21.1900221

**Published:** 2020-05-28

**Authors:** Paul P Schneider, Christel JAW van Gool, Peter Spreeuwenberg, Mariëtte Hooiveld, Gé A Donker, David J Barnett, John Paget

**Affiliations:** 1 School of Health and Related Research (ScHARR), University of Sheffield, Sheffield, United Kingdom; 2 Nivel (Netherlands Institute for Health Service Research), Utrecht, Netherlands; 3 School CAPHRI, Care and Public Health Research Institute, Maastricht University, Maastricht, Netherlands; 4 Faculty of Health, Medicine and Life Sciences, Maastricht University, Maastricht, Netherlands

**Keywords:** infectious diseases, influenza-like illness, surveillance, digital epidemiology, machine learning

## Abstract

**Background:**

Despite the early development of Google Flu Trends in 2009, standards for digital epidemiology methods have not been established and research from European countries is scarce.

**Aim:**

In this article, we study the use of web search queries to monitor influenza-like illness (ILI) rates in the Netherlands in real time.

**Methods:**

In this retrospective analysis, we simulated the weekly use of a prediction model for estimating the then-current ILI incidence across the 2017/18 influenza season solely based on Google search query data. We used weekly ILI data as reported to The European Surveillance System (TESSY)  each week, and we removed the then-last 4 weeks from our dataset. We then fitted a prediction model based on the then-most-recent search query data from Google Trends to fill the 4-week gap (‘Nowcasting’). Lasso regression, in combination with cross-validation, was applied to select predictors and to fit the 52 models, one for each week of the season.

**Results:**

The models provided accurate predictions with a mean and maximum absolute error of 1.40 (95% confidence interval: 1.09–1.75) and 6.36 per 10,000 population. The onset, peak and end of the epidemic were predicted with an error of 1, 3 and 2 weeks, respectively. The number of search terms retained as predictors ranged from three to five, with one keyword, ‘griep’ (‘flu’), having the most weight in all models.

**Discussion:**

This study demonstrates the feasibility of accurate, real-time ILI incidence predictions in the Netherlands using Google search query data.

## Introduction

Previous studies suggest that traditional disease surveillance systems could be complemented with information from online data sources [1-3]. The underlying premise is that nowadays, people, often turn to the Internet when they face health problems [4]. With influenza-like illness (ILI), individuals might search for information about symptoms, look for remedies or share messages on social media. All of these interactions leave digital footprints, which, when aggregated, could be harnessed to monitor disease activity [1]. In this way, online data streams could be used to support the timely detection of infectious disease outbreaks.

This hypothesis is not new, and in 2009, researchers at Google reported that their Flu Trends model was able to predict ILI activity in the United States (US) in real time, by monitoring millions of queries on their search engine [5]. The aim of Google Flu Trends was to bridge a 2-week lag in the reporting of ILI cases in the official surveillance statistics. Initially, the project appeared to provide accurate predictions and was expanded to cover 29 countries around the world. In 2012, however, the model’s performance deteriorated, and in early 2013, it overestimated the peak of the epidemic by more than 140%. The failure, and subsequent termination of Google Flu Trends, received a lot of media attention and sparked an intense debate about the limitations of big data in epidemiological research [3,6].

Since then, the number of scholarly articles published in the field of digital epidemiology has grown considerably [2,7]. The discipline is, nevertheless, in an early stage and should still be considered as being experimental.

Outside of the US, there has been little effort to investigate the value of online data sources for epidemiological purposes. Building on previous work [8], our study assessed whether online search queries could be used to predict the seasonal influenza epidemic in the Netherlands during the 2017/18 winter in real time.

## Methods

### Influenza-like illness data

Weekly data on consultations for ILI were collected through sentinel practices participating in the Nivel Primary Care Database [9]. The practices constitute a nationally representative group of 40 general practices in the Netherlands. The methodology is further described by Donker [10]. The data were available in real time and the ILI data for the last 4 weeks were only removed/assumed to be missing for the purpose of our study. For this study, we used preliminary ILI incidence data as reported to The European Surveillance System (TESSY) operated by the European Centre for Disease Prevention and Control [11]. Final ILI estimates can be retrieved from Nivel [9].

### Web search queries

Data on Google search queries were retrieved from Google Trends [12]. This online service provides information on how often a particular keyword was searched relative to the total search volume across various regions of the world. The granularity ranges from hourly to monthly time series data.

Potentially relevant search keywords were determined automatically, i.e. without being influenced by our judgment or expectations, using another Google Trends service, ‘find related searches’. Our starting point was the search term ‘griep’, the Dutch word for ‘flu’. We then retrieved the 25 most related search queries, i.e. keywords that Google users also searched for during the same session in which they searched ‘griep’. All keywords that contained a year were excluded because they were expected to be poor predictors of ILI rates in other years. For the remaining search terms, we used the R package gtrendsR [13] to download 5 years of weekly Google search query statistics, from week 33/2013 to week 31/2018, for the Netherlands.

### Modelling

We simulated the weekly use of a statistical model, based on Google search query data, for predicting the then current ILI incidence across the 2017/18 influenza season in the Netherlands. For this purpose, official ILI estimates for the then-latest 4 weeks, which included the then-current week, were removed from the dataset. Subsequently, a prediction model, solely based on the then most recent Google Trends data was used to predict the 4 weeks of missing data (‘Nowcasting’).

Based on visual inspection of the bivariate associations between Google searches and the ILI incidence, we decided to include square terms (n = 20) to account for nonlinearities. The set of predictors was further expanded by multiplying each predictor with all other predictors to account for one-level interactions (n = 190). Together with the original keywords (n = 20), a total of 230 variables were considered. Time dummy variables, seasonal effects or autoregressive terms were not considered. To identify and select the variables that are the best predictors of the ILI incidence, and to remove all other variables from the model, we used least absolute shrinkage and selection operator (lasso) regression [14] in combination with cross validation (CV).

To validate our modelling approach, we simulated the repetitive use of the prediction model during the 2017/18 influenza season. To do this, each week, the model was updated with the then-most recently available information: Google search query data up until the then-current week (week *w*) and the ILI incidence data up until 3 weeks earlier (*w−1* to *w−3*). The updated model was repetitively used to predict the ILI incidence for the then-current and the previous 3 weeks (*w* to *w−3*). Finally, predictions were compared with the observed values to assess the model’s performance.

### Automated analysis loop

The first 4 years of data (weeks 33/2013 to 30/2017) were used as training data only, while the analysis loop was run on the 52 weeks of the 2017/18 analysis period (weeks 31/2017 to 31/2018). At each week, the data were split into two parts; first, a training set, for which both the ILI and Google data were made available to the model, and second, a 4-week validation set (*w to w−3)*, for which only Google data were made available and for which the ILI data were removed/missing. This means, at the *ith* iteration of the loop, the analysis contained 207 + *i* weeks of training data (week 1 to 207 + *i)* and 4 weeks of validation data (week 207 + *i* + 1 to 207 + *i* + 4). Each week, a new model was built to predict the ILI incidence of the then-current week and the previous 3 weeks.

The model building process included the following steps. Dependent and independent variables were normalised and centred, with a mean of zero. The scaling for the training data was determined separately and then applied to the validation data to prevent information leaking from the validation to the training set. Variables with near zero variance were removed. Lasso regression in combination with CV was used to determine the optimal set of predictors and their regularised coefficients.

Lasso regression performs simultaneous variable selection and coefficient estimation. It imposes a penalty on the absolute values of the coefficients in the least squares estimation. In effect, less important parameters are shrunk towards zero and are excluded from the model, if their coefficients become zero. The model’s complexity is controlled by the penalty parameter λ.

In order to find the optimal value for λ, we used rolling forecast CV for time series, with fixed origin and expanding window. CV for time series is a variation of leave-*k*-out CV, which can be used to avoid the leakage of information from future to past observations. Similar to our automated analysis loop, CV for time series splits the data iteratively into a training set (the first *k* weeks) and a test or hold-out set (the subsequent 4 weeks). In the first CV iteration, a lasso regression model is fit on data from week 1 to *k* = 52 and its predictions are tested on hold-out data from week 53 to 56. The process then rolls forward, week-by-week, keeping the origin at week 1, and using an expanding number of weeks as training data with *k* = 52 + 1,  + 2,..., + *m*, whereby *m* increases with each iteration *i* of the outer analysis loop, with *m* = 207 − 52 + *i* . The prediction error over all *m* 4-week hold-out sets is then aggregated to assess how well the statistical model can predict new data points. At each iteration of the outer analysis loop, the inner CV loop is run for 100 values of the penalty parameter λ (ranging from 10^-8^ to 10^1/4^).

The λ*_i_* of the model with the lowest maximum absolute error in the CV hold-out sets was selected to fit the *ith* final model on 207 + *i* weeks of training data and to predict the ILI incidence for weeks 1 to 4 of the *ith* validation set ( i.e. weeks *w* to *w−3*). We used the lowest maximum, instead of the more common mean*,* absolute error as the criterion as it was considered more relevant in the context of ILI surveillance; models were selected as to minimise the worst-case scenario, i.e. a considerable over prediction, which could be falsely interpreted as the beginning of an epidemic.

For further information on variable selection, lasso regression and CV for time series, we suggest Heinze et al. [15], Kuhn [16] and Tashman [17].

### Model evaluation

We analysed the predictions of 52 lasso regression models, one for each week of the year. Each model provided four predicted values, corresponding to the 4 weeks of the validation sets (except the first/last three models which had shorter horizons). We refer to the prediction of the then-current week as week 4 prediction (i.e. 4 weeks since the last update with official ILI data), and to the predicted values for the previous three weeks as week 3 to 1 predictions.

We plotted the observed against the predicted ILI incidence values and assessed the performance of the statistical models over the validation period in terms of the mean absolute error (MAE). All values were back-transformed to their original scale and the accuracy of the week 1 to 4 predictions were evaluated separately. The 95% confidence intervals (CI) around mean estimates were bootstrapped using 10,000 resampling iterations. Prediction intervals were computed using the empirical non-parametric approach described by Lee and Scholtes [18], as the 2.5th and 97.5th quantile of the out-of-sample prediction errors. These intervals do not only capture the random variation in the data-generating process, but also the uncertainty in the model selection and potential misspecifications. They therefore provide robust estimates of the model fit. However, it is important to note that the prediction interval can only be computed retrospectively, after the models’ performances have been evaluated on the validation set; in other words, it cannot be known during the influenza season. We also reported the Pearson and Spearman correlation coefficients (r).

In addition, we assessed how accurately the models predicted the onset and peak of the season, and investigated which search query terms were retained as predictors in the 52 models. CIs around lasso regression coefficients were estimated conditional on the chosen final values of the tuning parameter λ [19].

### Source code and data availability

The R code for this study is provided under open, creative commons (CC) BY license and all data that were used for this study can be accessed online [20].

### Ethical statement

This study is based on publicly available secondary data and no personal identifiable information were used. Ethical approval was not required.

## Results

### Google search queries

We retrieved information on 26 search terms, ‘griep’ and the 25 most related keywords, from Google Trends (Table). Six terms were excluded from the analysis as they contained a year. For all other terms (n = 20), weekly search query statistics from 17 August 2013 (week 33/2013) to 4 August 2018 (week 31/2018) were downloaded.

**Table:** Dutch search terms retrieved from Google Trends and their English translation, Netherlands, 17 August 2013–4 August 2018 (n = 26 search terms)

Dutch search term	English translation
Griep	Flu
Symptomen	Symptoms
Symptomen griep	Symptoms flu
De griep	The flu
Griep 2018^a^	Flu 2018^a^
Griep koorts	Flu fever
Griep 2016^a^	Flu 2016^a^
Koorts	Fever
Tegen griep	Against flu
Griep hoe lang	Flu how long
Griep 2015^a^	Flu 2015^a^
Griep 2017^a^	Flu 2017^a^
Griep heerst	Flu going around
Symptomen griep 2018^a^	Symptoms flu 2018^a^
Verkoudheid	Common cold
Hoe lang duurt griep?	How long does flu last?
Ziek	Ill
Griep 2014^a^	Flu 2014^a^
Griep hoofdpijn	Flu headache
Griep wat te doen	Flu what to do
Heerst er griep	Is there flu going around
Griep zwanger	Flu pregnant
Verschijnselen griep	Symptoms flu
Griep kind	Flu child
Griep spierpijn	Flu muscle strain
Griep hoesten	Flu cough

^a^ Terms with a year in them were removed from further analysis because they were unlikely to be useful predictors in any other year.

**Figure 1:**
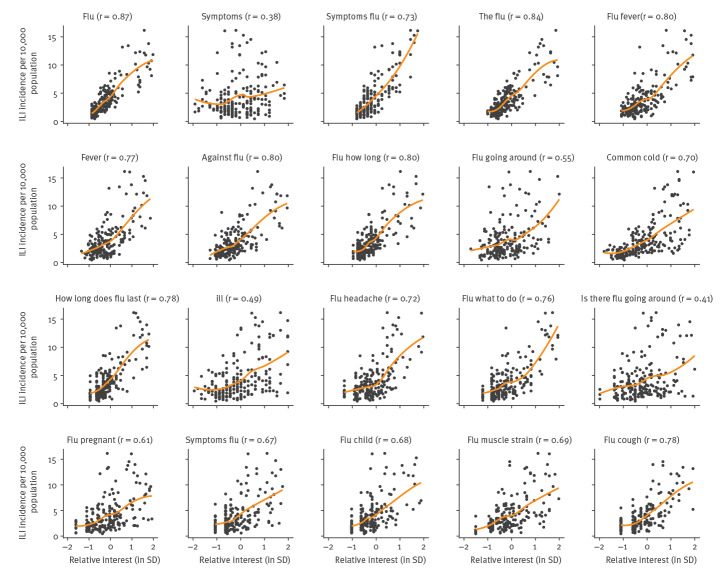
Bivariate associations between Google search terms and influenza-like illness incidence in the training dataset, Netherlands, weeks 33/2013–30/2017 (n = 20 search terms)

ILI: influenza-like illness; SD: standard deviation.

Overall, there was a high correlation between the search query statistics and the ILI incidence in the initial training dataset (week 33/2013 to week 30/2017), with a mean correlation coefficient of 0.69 (Figure 1). The lowest correlation was observed for the term ‘symptomen’ (r: 0.38), and the highest for the term ‘griep’ (r: 0.87). Between search terms, the degree of collinearity was also high, with an average correlation coefficient of 0.70 (minimum: 0.24; maximum: 0.97).

Provided are the Pearson correlation coefficients (r) and locally-estimated scatterplot smoothing (LOESS) curves fitted to data points of the initial training period (weeks 33/2013 to 30/2017). Search terms in Dutch can be found in the Table.

### Real-time influenza-like illness incidence prediction models

**Figure 2:**
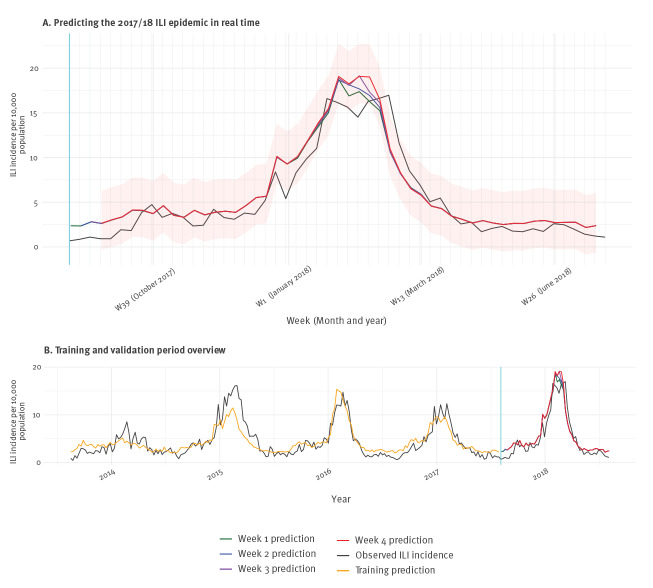
Time series plot showing observed influenza-like illness incidence against predictions of 52 final lasso regression models, weeks 31/2017–31/2018 (A) and overview of training and validation, weeks 33/2013–31/2018 (B), Netherlands

We simulated the weekly use of a real-time prediction model during the 52 weeks of the analysis period. At each week, a new prediction model was built to estimate the ILI incidence of the current week (week 4) and the previous three weeks (weeks 3 to 1). Figure 2 shows the values of these week 1 to 4 predictions separately against the observed ILI incidence, alongside the 95% prediction interval for week 4 predictions.

ILI: influenza-like illness.

For the validation period (top), each model provides estimates for the week in which it was run (week 4) and the previous 3 weeks (week 3 to 1). The red shaded area shows the empirical 95% prediction interval for week 4 predictions. In addition, predicted values within the training set are provided for the first lasso regression model (bottom).

The MAE for ILI incidence predictions of weeks 1, 2, 3 and 4 across the 52 final lasso regression models were 1.31 (95% CI: 1.03–1.62), 1.35 (95% CI: 1.07–1.68), 1.38 (95% CI: 1.07–1.73) and 1.40 (95% CI: 1.09–1.75). The corresponding non-parametric 95% prediction intervals ranged from −2.98 to 2.78 for week 1; from −3.02 to 3.00 for week 2; from −3.07 to 3.59 for week 3; and from −3.01 to 3.65 for week 4 (Figure 2). Pearson correlation between observed and predicted values varied between 0.95 and 0.94, and Spearman correlation coefficients were 0.90 for all 4 weeks.

The error was generally low before the start of the seasonal epidemic in the Netherlands in week 50/2017 [21], but it increased during the onset, and especially during the epidemic peak, when the highest prediction error (6.36) was registered (week 10/2018). After weeks 9/ and 10/2018, the incidence was underpredicted by the models.

The model’s MAE in the validation period was slightly lower than the MAE observed in the CV hold-out sets (CV MAE for 1,2,3 and 4 predictions were 1.49, 1.55, 1.63 and 1.68). The maximum absolute error was markedly lower in the CV hold out sets (1.72).

The bottom plot in Figure 2 provides an overview of the entire 5-year observation period. The vertical blue line separates the training (left) from the validation period (right). For comparative purposes, the predicted values for the training period of the first prediction model (run in week 31/2017) are provided (first model training MAE: 1.32; maximum error: 7.18). The figure also illustrates the seasonality of influenza epidemics (black line). The seasonal epidemic in 2017/18 had a slightly higher intensity and lasted longer than average, but was otherwise not exceptional.

### Temporal aspects of influenza-like illness incidence predictions

**Figure 3:**
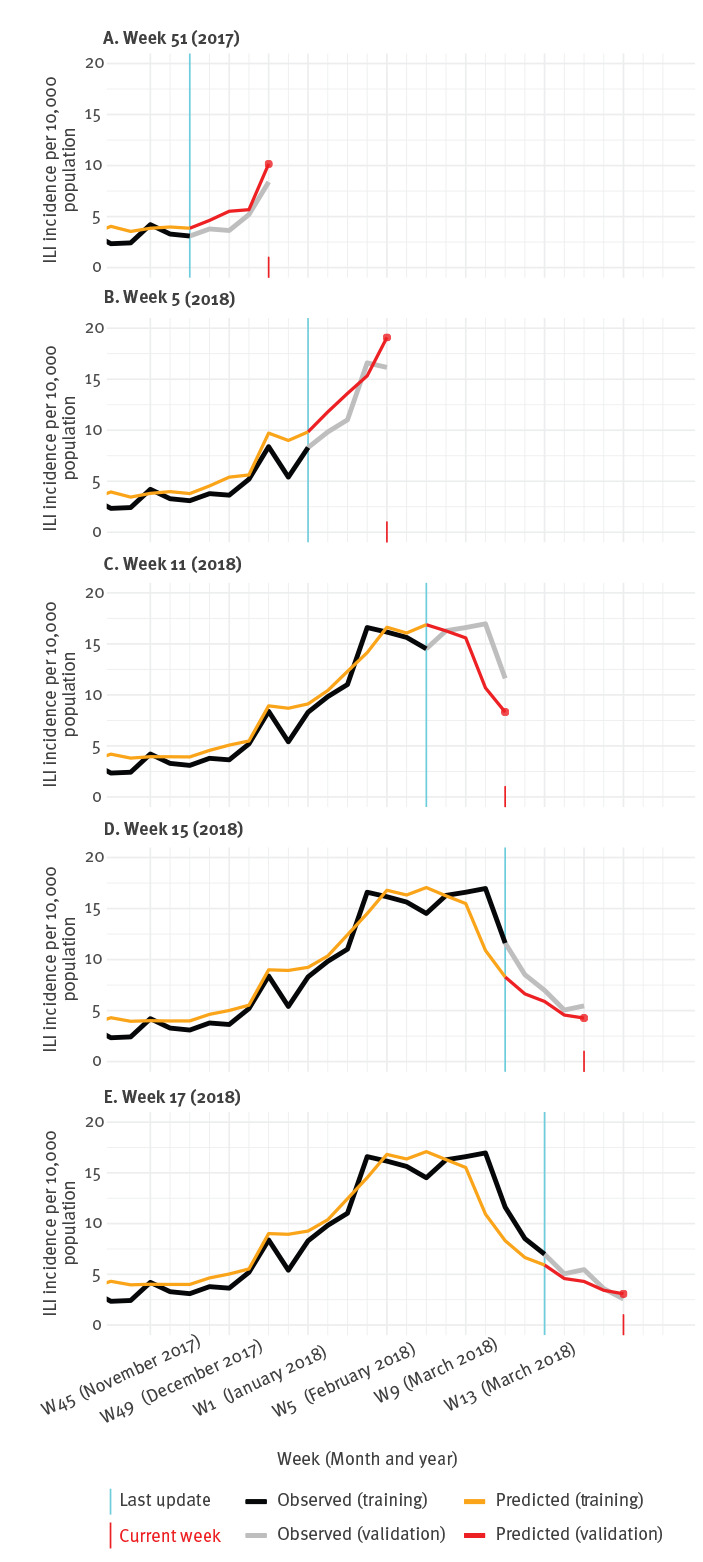
Observed vs predicted influenza-like illness incidence at five time points (A–E), Netherlands, influenza season 2017/18

From the visual presentation in Figure 2, it might be difficult to assess what information was available at which week. To illustrate the temporal dynamics of the ILI prediction model, Figure 3 shows model results at five different points in time.

ILI: influenza-like illness.

The plots illustrate what information the prediction models would have provided at these five particular points in time (indicated by the red dot and vertical line), if they had been used. The week for which the last official ILI incidence data were made available is marked by the vertical cyan line.

The model would have indicated the onset of the season 1 week ahead of the sentinel surveillance data (Panel A: observed onset was week 50/2017, predicted onset was week 49 /2017). The peak of the season was predicted in week 07/2018 (Panel B), while the observed peak was biphasic with the highest incidence (16.97/10,000 population) in week 10/2018 and the second highest (16.6/10,000 population) in week 04/2018 (Panel C and D). The end of the season, i.e. when ILI incidence falls below 5.1 per 10,000 population for 2 consecutive weeks, was predicted in week 14/2018, and observed in week 16/2018 (Panel E).

Visual inspection indicates that the predictions generally appeared to be ahead of the actual ILI incidence. Throughout the validation period, week 4 predictions were forecasting the ILI incidence of the coming week slightly more accurately (MAE: 1.11) than predicting the current week (MAE: 1.40).

### Model specifications

**Figure 4:**
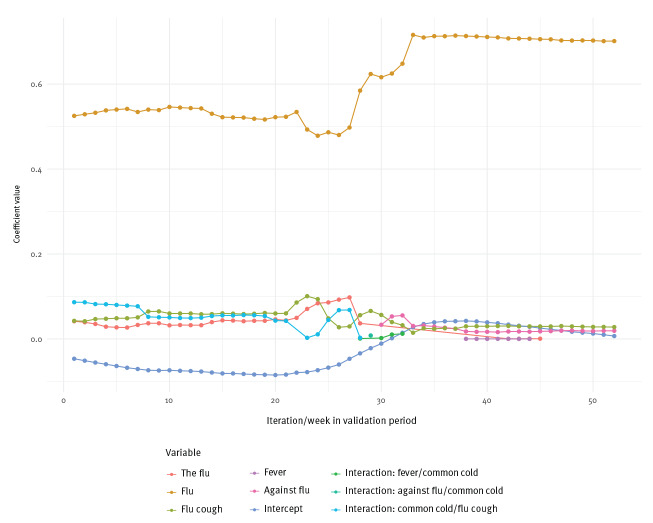
Predictors retained in the final lasso regression models throughout the 52 iterations, Netherlands, weeks 31/2017–31/2018

Figure 4 provides an overview of the 52 weekly sets of predictors and their coefficients used in the final prediction models. During the validation period, the number of variables that were retained in the models as predictors ranged from 3 to 5. Even though the validity of statistical inference after performing model selection is limited, it is interesting to note that one predictor, ‘griep’ (‘flu’) had by far the most weight in all models, especially after week 32 of the validation period, which was week 11/2018. It is also interesting to note that the coefficients for all other predictors had wide confidence intervals and were not significantly different from zero (see Supplementary Figure S1 and Table S2).

Lasso coefficients with a value of zero are not shown.

Search terms in Dutch can be found in the Table.

## Discussion

Our study demonstrates that a statistical model based on online search queries could have been used to monitor the ILI incidence in the Netherlands during the 2017/18 influenza season. Assuming a delay of 4 weeks between the incidence and the reporting of ILI cases, our model predicted the epidemic in real time with high accuracy: the onset, peak and end of the epidemic would have been identified with 1 to 3 weeks difference.

This investigation provides an accessible but rigorous case study in digital epidemiology. The modelling steps are tractable and computationally economical such that the source code can be modified and applied to other settings/countries and other infectious diseases or health conditions with a seasonal pattern, e.g. hay fever, allergic asthma. The full source code and data are provided under open license to encourage the application of this method to other countries and to other areas of epidemiological research.

A notable feature of our study is the week-by-week simulation of the prediction model. We built 52 models, each of which was validated on 4 weeks of data (which were later used for fitting subsequent models). The iterative analysis loop allowed us to set a realistic framework for investigating our research question. Our results reflect how well a model would have performed and what information it would have provided if it was used during the 2017/18 influenza season. The loop structure also enabled us to continuously update the model, as suggested by previous research [6,22], to prevent deterioration of performance. Each week, we re-fitted the prediction model using the most recently available ILI data from 4 weeks ago. In addition, we also repetitively applied CV to select the momentarily optimal set of predictors; interestingly, most retained variables only changed marginally over time, and one predictor (‘griep’) had by far the highest weight in all prediction models. However, our models were designed for the purpose of prediction, not explanation, and results with regard to individual predictors should thus be interpreted with caution. Our evaluation of the model showed that the week 1 to week 4 predictions were highly consistent and discrepancies were only observed during the peak of the influenza season.

When preparing this project, we assessed a number of different data sources, e.g. Google Trends and Wikipedia page visits [8], and chose Google Trends [12] to be the most advantageous for our project. This is because the Google Trends service is publicly available, easy to use and is understood to cover the online search behaviour of the majority of people in the Netherlands. However, its use comes with certain limitations that should be considered when interpreting findings or applying this methodology. Using the public application programming interface (API), weekly data can only be retrieved for periods of less than 5 years, which sets a boundary on the observation period. There is also a quota for the number of search requests for an individual per session that limits the amount of predictor data that can be downloaded. Moreover, we cannot rule out that data was leaked from future to past observations since we retrieved all data after the end of the season. If the data were retrieved each week during the season, results could have been different [23].

We provide an accurate and computationally efficient approach to model the use of web search queries to monitor ILI rates over time. To further improve predictive performance, future studies should consider the following strategies that have been successfully applied in previous disease prediction models: (i) using other online data sources, e.g. Wikipedia page views, Twitter activity [20,22], (ii) conducting, more extensive data pre-processing, e.g. principal component analysis [24,25], (iii) applying alternative statistical models, e.g. smoothing splines, ensemble methods [7,25], or (iv) considering different combinations and interaction terms between predictors and (v) additional predictors besides online data, including seasonal and autoregressive terms [26]. In this study, seasonal and autoregressive terms were not considered, not only because it would have been computationally expensive to include those in the CV loop, but also because determining the added value of including Google search queries as variables in the prediction model would then have been more difficult. 

Our results are comparable to previous studies from European countries. Valdivia et al. [27] used historic data from the now terminated Google Flu Trends project and compared the predicted ILI rates against sentinel surveillance estimates across the 13 European countries during the 2009/10 influenza A(H1N1) pandemic. They found high correlations between predicted and observed ILI rates, with Spearman coefficients ranging from 0.72 in Poland to 0.94 in Germany. For the Netherlands, the authors reported a correlation of 0.86, which is slightly lower than what we found in our study (0.90). More recently, Samaras et al. [28] studied the association between ILI incidence and influenza-related Google search queries in Greece and Italy in 2011 and 2012. They found Pearson correlation coefficients between 0.83 for Greece and 0.98 for Italy. It should be noted, however, that these figures are based on a retrospective analysis of the data and, unlike in our study, the results were not validated on a test dataset. Moreover, correlations are not based on the absolute differences between the predicted and observed values and might therefore generate conflicting or even misleading results. Future studies should report measures of absolute differences, such as MAE, to enable appropriate comparison of predictive performances.

Numerous other studies, mostly from the US have aimed to predict ILI incidence rates from online data, using various data sources and applied an array of different methods [2,7]. Unfortunately, many of the published studies suffer from methodological limitations, such as the use of inappropriate outcome measures, e.g. correlations; the absence of a rigorous validation method, e.g. using a single dataset to fit a model and evaluate its predictions; or insufficient reporting, which does not allow for replication of results. Tabataba et al. [29] and Generous et al. [30] have published in-depth discussions of these points.

In the Netherlands, there is no justification to monitor ILI through internet search analyses as ILI data, including virological information, are collected from sentinel practices in near real time. However, during week 52/2017 and 1/2018, we made an interesting observation: sentinel surveillance data indicated a temporary drop in ILI incidence, but the signal was unlikely to have been caused by a decrease in the actual number of ILI cases, but rather by low healthcare utilisation and/or changes in doctors’ working hours during the Christmas and New Year holiday period. For these 2 weeks, it could be argued that our prediction model could have usefully complemented the sentinel surveillance system.

Further potential applications of digital epidemiology methods include the provision of supportive, low-cost, online surveillance in countries with limited resources that, for example, report data more slowly than the Netherlands (which does so on a weekly basis) or that do not have disease surveillance coverage of all regions or an early warning system for pandemic outbreaks [1]. However, before these novel methods can be applied in routine practice, they need to be thoroughly evaluated and their value has to be unequivocally determined. More research is needed to better understand where, when and how online surveillance can complement established systems.

Prediction models need to demonstrate that they provide accurate and reliable estimates. It is especially important to avoid false alarms that could, for example, be caused by ILI-related news reports. If a surveillance model cannot differentiate between news-related and symptom-related ILI searches, any increase in ILI-related search activity could trigger a warning that might then lead to unnecessary public anxiety and economic costs.

It is also important to note that the prediction model we used in this study was designed to accurately predict ILI incidence rates, not to asses which factors best explain it [31]. With high multicollinearity between predictors and after performing parameter selection before coefficient estimation, the ability to make sound inferences about individual variables coefficients is very limited. Further quantitative, as well as qualitative studies, are required to better understand the online health information seeking behaviour of individuals with and without ILI.

Another limitation is that most, if not all of the online data that is relevant for building prediction models, e.g. online searches and social media activity, are owned by private companies. Those companies could change the methods of data collection, processing or provision at any point in time without any advance notice. These challenges must be recognised, but they should not discourage further studies and developments in the field.

## Conclusions

Our study demonstrates that a prediction model based on online search queries could have predicted the 2017/18 ILI epidemic in the Netherlands in real time. The intensity of the epidemic, as well as its onset, peak and end were estimated with reasonable accuracy. The value of using online surveillance methods to complement traditional disease surveillance systems in Europe, and beyond, including for the current coronavirus disease (COVID-19) pandemic, should be further explored.

## Supplementary Data

371000 Supplementary Material
