# Supplementary material for: Using web search queries to monitor influenza-like illness: an exploratory retrospective analysis, Netherlands, 2017/18 influenza season
Source: Euro Surveill. 2020 May 28;25(21):1900221. doi: 10.2807/1560-7917.ES.2020.25.21.1900221 (PMC7268271; doi:10.2807/1560-7917.ES.2020.25.21.1900221)
Supplement: Supplementary Material [file 19-00221_SCHNEIDER_Supplementary_Material.pdf]

Supplementary Material

Disclaimer

This supplementary material is hosted by Eurosurveillance as supporting information alongside the article 'Using web search queries to monitor influenza-like illness: an exploratory retrospective analysis, Netherlands, 2017/18 influenza season' on behalf of the authors who remain responsible for the accuracy and appropriateness of the content. The same standards for ethics, copyright, attributions and permissions as for the article apply. Supplements are not edited by Eurosurveillance and the journal is not responsible for the maintenance of any links or email addresses provided therein.

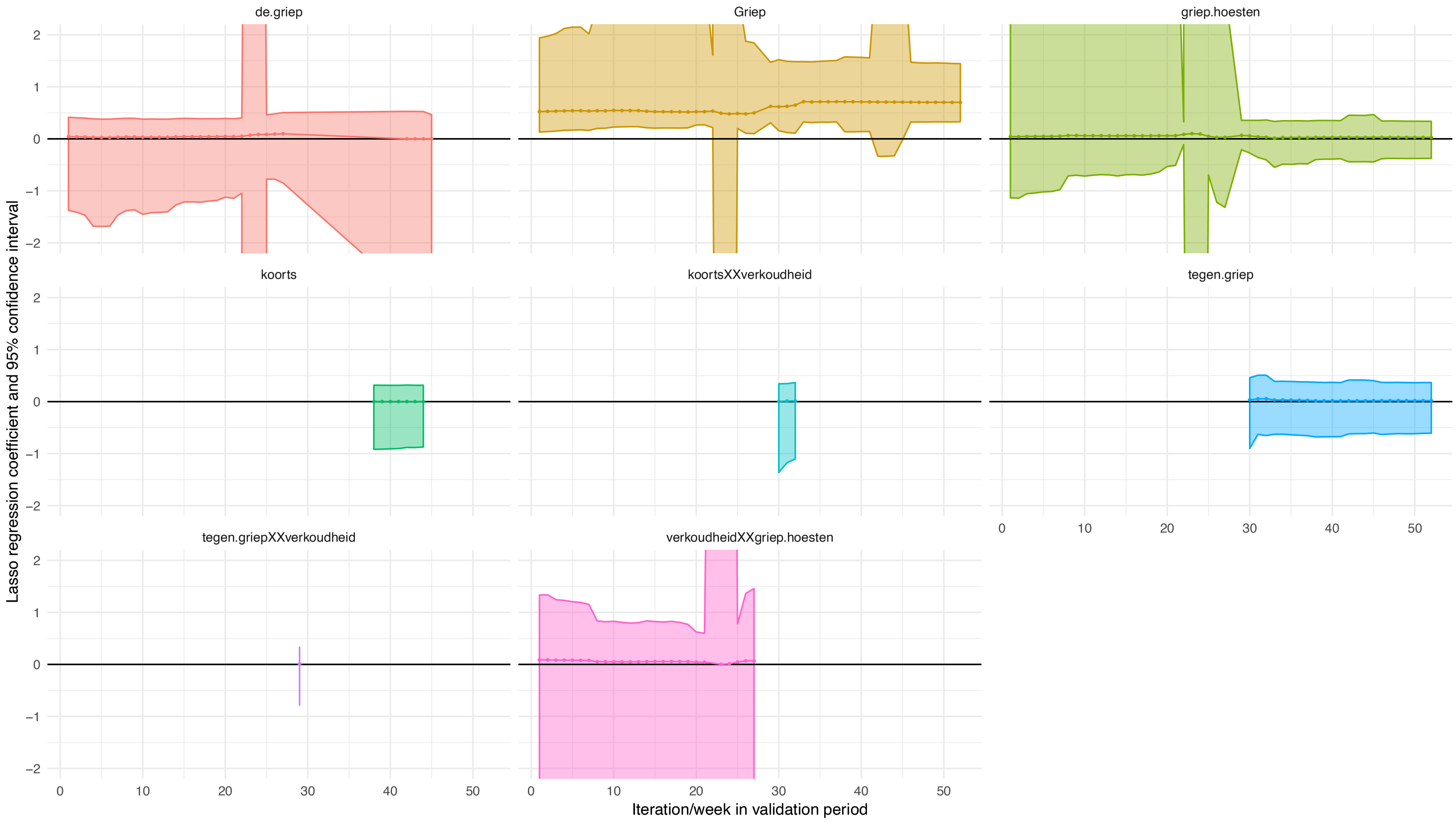

Figure S1: Lasso regression coefficients and 95% confidence intervals (CI), conditional on final hyperparameter lambda. Y axis is limited to values <2 and > -2.

Table S2: Lasso regression coefficients and 95% confidence intervals (CI), conditional on final hyperparameter lambda.

step/ week	Variable	Coefficient point estimate	95% CI lower bound	95% CI upper bound	p value
**1**	Griep	0,525	0,129	1,941	0,022
**1**	de griep	0,042	-1,374	0,414	0,657
**1**	griep hoesten	0,042	-1,134	2,376	0,327
**1**	verkoudheidXXgriep.hoesten	0,087	-3,174	1,333	0,667
**2**	Griep	0,529	0,139	1,973	0,021
**2**	de griep	0,039	-1,411	0,405	0,665
**2**	griep hoesten	0,042	-1,141	2,377	0,328
**2**	verkoudheidXXgriep.hoesten	0,086	-3,175	1,337	0,667
**3**	Griep	0,532	0,149	2,024	0,019
**3**	de griep	0,035	-1,468	0,399	0,676
**3**	griep hoesten	0,047	-1,056	2,521	0,303
**3**	verkoudheidXXgriep.hoesten	0,082	-3,382	1,244	0,690
**4**	Griep	0,538	0,164	2,121	0,017
**4**	de griep	0,029	-1,681	0,384	0,697
**4**	griep hoesten	0,047	-1,040	2,530	0,299
**4**	verkoudheidXXgriep.hoesten	0,082	-3,392	1,230	0,693
**5**	Griep	0,540	0,168	2,147	0,017
**5**	de griep	0,027	-1,683	0,379	0,701
**5**	griep hoesten	0,049	-1,020	2,571	0,293
**5**	verkoudheidXXgriep.hoesten	0,080	-3,453	1,205	0,699
**6**	Griep	0,542	0,173	2,148	0,016
**6**	de griep	0,027	-1,679	0,378	0,702
**6**	griep hoesten	0,048	-1,013	2,624	0,288
**6**	verkoudheidXXgriep.hoesten	0,079	-3,529	1,191	0,705
**7**	Griep	0,534	0,162	2,020	0,017
**7**	de griep	0,033	-1,462	0,389	0,680
**7**	griep hoesten	0,051	-0,978	2,670	0,279
**7**	verkoudheidXXgriep.hoesten	0,077	-3,596	1,154	0,713
**8**	Griep	0,540	0,201	2,537	0,014
**8**	de griep	0,037	-1,379	0,396	0,665
**8**	griep hoesten	0,064	-0,713	4,084	0,183
**8**	verkoudheidXXgriep.hoesten	0,052	-5,616	0,836	0,815
**9**	Griep	0,539	0,205	2,550	0,013
**9**	de griep	0,037	-1,364	0,394	0,663
**9**	griep hoesten	0,065	-0,700	4,101	0,180
**9**	verkoudheidXXgriep.hoesten	0,051	-5,636	0,820	0,817
**10**	Griep	0,546	0,227	2,619	0,011
**10**	de griep	0,032	-1,452	0,378	0,682
**10**	griep hoesten	0,060	-0,716	4,257	0,180
**10**	verkoudheidXXgriep.hoesten	0,051	-5,858	0,828	0,820
**11**	Griep	0,545	0,231	2,680	0,011
**11**	de griep	0,033	-1,420	0,381	0,678
**11**	griep hoesten	0,060	-0,699	4,385	0,174
**11**	verkoudheidXXgriep.hoesten	0,049	-6,039	0,808	0,826
**12**	Griep	0,543	0,235	2,678	0,011
**12**	de griep	0,032	-1,414	0,379	0,678
**12**	griep hoesten	0,060	-0,687	4,382	0,172

**12**	verkoudheidXXgriep.hoesten	0,049	-6,039	0,794	0,827
**13**	Griep	0,543	0,234	2,656	0,010
**13**	de griep	0,032	-1,403	0,377	0,677
**13**	griep hoesten	0,058	-0,694	4,340	0,174
**13**	verkoudheidXXgriep.hoesten	0,050	-5,973	0,802	0,825
**14**	Griep	0,530	0,212	2,431	0,012
**14**	de griep	0,039	-1,274	0,387	0,651
**14**	griep hoesten	0,058	-0,713	3,940	0,187
**14**	verkoudheidXXgriep.hoesten	0,054	-5,390	0,837	0,810
**15**	Griep	0,522	0,206	2,384	0,012
**15**	de griep	0,044	-1,213	0,393	0,639
**15**	griep hoesten	0,060	-0,690	3,882	0,184
**15**	verkoudheidXXgriep.hoesten	0,055	-5,297	0,824	0,810
**16**	Griep	0,522	0,209	2,382	0,012
**16**	de griep	0,043	-1,211	0,391	0,639
**16**	griep hoesten	0,059	-0,687	3,879	0,184
**16**	verkoudheidXXgriep.hoesten	0,055	-5,290	0,815	0,811
**17**	Griep	0,521	0,208	2,325	0,012
**17**	de griep	0,042	-1,218	0,386	0,643
**17**	griep hoesten	0,058	-0,697	3,762	0,188
**17**	verkoudheidXXgriep.hoesten	0,056	-5,123	0,828	0,806
**18**	Griep	0,518	0,207	2,337	0,012
**18**	de griep	0,043	-1,196	0,388	0,638
**18**	griep hoesten	0,059	-0,677	3,785	0,185
**18**	verkoudheidXXgriep.hoesten	0,056	-5,155	0,808	0,809
**19**	Griep	0,517	0,211	2,406	0,011
**19**	de griep	0,043	-1,183	0,386	0,637
**19**	griep hoesten	0,061	-0,639	3,929	0,175
**19**	verkoudheidXXgriep.hoesten	0,054	-5,362	0,767	0,818
**20**	Griep	0,522	0,265	3,141	0,008
**20**	de griep	0,046	-1,121	0,392	0,622
**20**	griep hoesten	0,060	-0,531	5,491	0,133
**20**	verkoudheidXXgriep.hoesten	0,043	-7,559	0,623	0,865
**21**	Griep	0,523	0,269	3,188	0,008
**21**	de griep	0,044	-1,144	0,387	0,629
**21**	griep hoesten	0,060	-0,512	5,594	0,130
**21**	verkoudheidXXgriep.hoesten	0,043	-7,704	0,600	0,868
**22**	Griep	0,534	0,215	1,610	0,007
**22**	de griep	0,049	-1,046	0,400	0,601
**22**	griep hoesten	0,086	-0,110	0,325	0,160
**23**	Griep	0,493	-22,543	Inf	0,000
**23**	de griep	0,071	-Inf	19,698	1,000
**23**	griep hoesten	0,101	-20,721	Inf	0,000
**23**	verkoudheidXXgriep.hoesten	0,003	-Inf	25,259	0,000
**24**	Griep	0,478	-22,616	Inf	0,000
**24**	de griep	0,084	-Inf	19,731	1,000
**24**	griep hoesten	0,094	-20,852	Inf	0,000
**24**	verkoudheidXXgriep.hoesten	0,011	-Inf	25,419	0,000
**25**	Griep	0,487	0,199	2,914	0,014
**25**	de griep	0,086	-0,775	0,460	0,497
**25**	griep hoesten	0,048	-0,693	4,953	0,164
**25**	verkoudheidXXgriep.hoesten	0,045	-6,834	0,779	0,841
**26**	Griep	0,480	0,108	1,876	0,025
**26**	de griep	0,092	-0,776	0,481	0,488

**26**	griep hoesten	0,027	-1,218	2,846	0,311
**26**	verkoudheidXXgriep.hoesten	0,068	-3,847	1,365	0,700
**27**	Griep	0,498	0,098	1,845	0,027
**27**	de griep	0,098	-0,848	0,505	0,498
**27**	griep hoesten	0,029	-1,317	2,846	0,325
**27**	verkoudheidXXgriep.hoesten	0,068	-3,793	1,459	0,687
**29**	Griep	0,624	0,307	1,471	0,002
**29**	griep hoesten	0,066	-0,208	0,355	0,244
**29**	tegen.griepXXverkoudheid	0,008	-0,785	0,331	0,573
**30**	Griep	0,616	0,155	1,522	0,017
**30**	tegen griep	0,033	-0,900	0,460	0,575
**30**	griep hoesten	0,056	-0,274	0,356	0,299
**30**	koortsXXverkoudheid	0,002	-1,362	0,343	0,684
**31**	Griep	0,625	0,120	1,491	0,022
**31**	tegen griep	0,053	-0,630	0,507	0,463
**31**	griep hoesten	0,040	-0,358	0,355	0,359
**31**	koortsXXverkoudheid	0,010	-1,176	0,346	0,665
**32**	Griep	0,648	0,110	1,483	0,025
**32**	tegen griep	0,055	-0,653	0,508	0,468
**32**	griep hoesten	0,032	-0,405	0,363	0,386
**32**	koortsXXverkoudheid	0,012	-1,103	0,367	0,642
**33**	Griep	0,716	0,325	1,483	0,002
**33**	tegen griep	0,030	-0,627	0,390	0,478
**33**	griep hoesten	0,014	-0,549	0,334	0,483
**34**	Griep	0,710	0,312	1,479	0,002
**34**	tegen griep	0,031	-0,626	0,391	0,476
**34**	griep hoesten	0,025	-0,487	0,347	0,441
**35**	Griep	0,713	0,320	1,490	0,002
**35**	tegen griep	0,029	-0,639	0,387	0,484
**35**	griep hoesten	0,024	-0,492	0,346	0,446
**36**	Griep	0,713	0,319	1,499	0,002
**36**	tegen griep	0,026	-0,646	0,381	0,489
**36**	griep hoesten	0,025	-0,478	0,348	0,437
**37**	Griep	0,714	0,326	1,505	0,002
**37**	tegen griep	0,024	-0,655	0,378	0,497
**37**	griep hoesten	0,024	-0,481	0,343	0,441
**38**	Griep	0,713	0,135	1,576	0,021
**38**	koorts	0,000	-0,917	0,316	0,621
**38**	tegen griep	0,018	-0,679	0,373	0,511
**38**	griep hoesten	0,030	-0,399	0,352	0,388
**39**	Griep	0,712	0,135	1,571	0,021
**39**	koorts	0,000	-0,914	0,315	0,620
**39**	tegen griep	0,017	-0,676	0,368	0,512
**39**	griep hoesten	0,030	-0,391	0,353	0,384
**40**	Griep	0,711	0,140	1,566	0,021
**40**	koorts	0,000	-0,908	0,314	0,620
**40**	tegen griep	0,017	-0,674	0,371	0,510
**40**	griep hoesten	0,030	-0,390	0,352	0,383
**41**	Griep	0,710	0,139	1,556	0,021
**41**	koorts	0,000	-0,902	0,313	0,618
**41**	tegen griep	0,016	-0,673	0,366	0,511
**41**	griep hoesten	0,030	-0,382	0,350	0,379
**42**	Griep	0,708	-0,336	3,645	0,107
**42**	de griep	0,000	-2,963	0,528	0,766

**42**	koorts	0,000	-0,881	0,319	0,611
**42**	tegen griep	0,017	-0,621	0,416	0,476
**42**	griep hoesten	0,030	-0,442	0,454	0,404
**43**	Griep	0,708	-0,336	3,620	0,106
**43**	de griep	0,000	-2,945	0,527	0,766
**43**	koorts	0,000	-0,884	0,314	0,614
**43**	tegen griep	0,017	-0,616	0,415	0,474
**43**	griep hoesten	0,030	-0,440	0,452	0,405
**44**	Griep	0,707	-0,326	3,607	0,106
**44**	de griep	0,000	-2,930	0,525	0,765
**44**	koorts	0,000	-0,871	0,315	0,611
**44**	tegen griep	0,017	-0,615	0,414	0,473
**44**	griep hoesten	0,030	-0,439	0,451	0,405
**45**	Griep	0,706	-0,018	3,810	0,052
**45**	de griep	0,000	-3,121	0,465	0,784
**45**	tegen griep	0,018	-0,603	0,403	0,470
**45**	griep hoesten	0,029	-0,444	0,467	0,406
**46**	Griep	0,705	0,322	1,473	0,002
**46**	tegen griep	0,018	-0,632	0,369	0,493
**46**	griep hoesten	0,029	-0,382	0,343	0,384
**47**	Griep	0,703	0,324	1,461	0,002
**47**	tegen griep	0,019	-0,623	0,368	0,489
**47**	griep hoesten	0,030	-0,375	0,344	0,380
**48**	Griep	0,703	0,324	1,458	0,002
**48**	tegen griep	0,020	-0,615	0,370	0,486
**48**	griep hoesten	0,029	-0,377	0,343	0,382
**49**	Griep	0,703	0,327	1,460	0,002
**49**	tegen griep	0,019	-0,619	0,365	0,490
**49**	griep hoesten	0,029	-0,379	0,339	0,384
**50**	Griep	0,703	0,326	1,456	0,002
**50**	tegen griep	0,018	-0,617	0,365	0,490
**50**	griep hoesten	0,028	-0,378	0,338	0,385
**51**	Griep	0,701	0,325	1,448	0,002
**51**	tegen griep	0,019	-0,609	0,367	0,487
**51**	griep hoesten	0,028	-0,377	0,337	0,384
**52**	Griep	0,701	0,329	1,446	0,002
**52**	tegen griep	0,019	-0,609	0,366	0,486
**52**	griep hoesten	0,028	-0,376	0,337	0,384
